# miTALOS v2: Analyzing Tissue Specific microRNA Function

**DOI:** 10.1371/journal.pone.0151771

**Published:** 2016-03-21

**Authors:** Martin Preusse, Fabian J. Theis, Nikola S. Mueller

**Affiliations:** 1 Institute of Computational Biology, Helmholtz Zentrum München, German Research Center for Environmental Health, Neuherberg, Germany; 2 Institute of Diabetes and Regeneration Research, Helmholtz Zentrum München, German Research Center for Environmental Health, Neuherberg, Germany; 3 Institute for Mathematical Sciences, Technische Universität München, Munich, Germany; National Institute of Technology, Rourkela, INDIA

## Abstract

MicroRNAs are involved in almost all biological processes and have emerged as regulators of signaling pathways. We show that miRNA target genes and pathway genes are not uniformly expressed across human tissues. To capture tissue specific effects, we developed a novel methodology for tissue specific pathway analysis of miRNAs. We incorporated the most recent and highest quality miRNA targeting data (TargetScan and StarBase), RNA-seq based gene expression data (EBI Expression Atlas) and multiple new pathway data sources to increase the biological relevance of the predicted miRNA-pathway associations. We identified new potential roles of miR-199a-3p, miR-199b-3p and the miR-200 family in hepatocellular carcinoma, involving the regulation of metastasis through MAPK and Wnt signaling. Also, an association of miR-571 and Notch signaling in liver fibrosis was proposed. To facilitate data update and future extensions of our tool, we developed a flexible database backend using the graph database neo4j. The new backend as well as the novel methodology were included in the updated miTALOS v2, a tool that provides insights into tissue specific miRNA regulation of biological pathways. miTALOS v2 is available at http://mips.helmholtz-muenchen.de/mitalos.

## Introduction

MicroRNAs (miRNAs) are short, non-coding RNAs that regulate gene expression post transcriptionally through binding to a target mRNA. They are predicted to target hundreds of genes in mammals and most genes are thought to be regulated by miRNAs [1]. Consequently, most biological processes involve miRNAs and miRNA-mediated control of gene expression.

Functional analysis of miRNAs depends on accurate identification of gene targets in a given biological context [2]. Since there is no comprehensive catalogue of tissue and cell type specific miRNA-mRNA interactions, computational target prediction tools are still widely used. Although these prediction tools have improved in accuracy, they still suffer from large numbers of false-positive miRNA-mRNA interactions [2]. Recently, biochemical methods using sequencing of target RNA isolated after UV crosslinking and immunoprecipitation of Ago/miRNA complexes (CLIP-seq) were developed [3,4]. They produce a map of miRNA binding sites on their target mRNAs. CLIP-seq data is collected in the StarBase database [5], providing a constantly growing resource of experimentally supported interactions. While these experimental methods increase the specificity of miRNA target data, their explanatory power is limited due to differences in experimental procedures and lack of reproducibility [6]. Moreover, all human data sets in StarBase were measured in immortalized cell lines (HEK293, HeLa) and not in primary tissue.

Next to limitations of *in-silico* and experimental gene target identification, miRNA-mediated regulation suggested by *in-vitro* and cell culture experiments is often not supported by *in-vivo* validation studies [7]. This can be partly explained by the fact that most miRNAs show only limited effects on the level of individual target mRNAs under physiological conditions [8]. In addition, target prediction and CLIP-seq studies demonstrated that most mRNAs are regulated by multiple miRNAs [9–11]. Thus, the down-regulation of a target gene depends on the combined effect of multiple miRNAs. And analysis of individual miRNA-mRNA interactions is not sufficient to explain the regulatory role of miRNAs in biological process.

Computational approaches often perform a pathway analysis to increase the explanatory power of target gene sets and to circumvent the shortcomings in targeting data. They use the complete set of miRNA target genes and pathway genes to associate miRNAs to biological pathways as an indication of their biological function. In doing so they do not account for the characteristic tissue expression signature of mammalian genes [12] and thus disregard tissue specific effects of miRNAs. Indeed, miRNAs were shown to facilitate tissue specificity of gene regulation [13]. Moreover, other pathway analysis tools such as DIANA mirPath rely on target prediction only and do not use CLIP-seq based target data [14].

Tissue-specific gene expression data can be obtained using next-generation sequencing of RNA (RNA-seq). The EBI Expression Atlas [15] collects highly curated gene expression data sets and also includes baseline expression data for healthy tissue or untreated cell lines in various organisms. Baseline expression describes the abundance of a gene and is extracted from large-scale expression studies such as ENCODE cell lines.

We developed a novel pathway analysis methodology leveraging this high-quality tissue expression data in order to predict miRNA function. We used our new methodology to first analyze the role of miRNAs in hepatocellular carcinoma and identified the liver-specific effect of miR-199a/b-3p on pathways associated with proliferation and cell migration, a novel function that a recent study proposed. We next dissected the individual functions of the two genomic clusters of the miR-200 family and found hints to new signaling relationships, which were studied in other tissues and cell culture but not yet in liver cancer. We finally extended our analysis to liver fibrosis, which is in general less well studied than liver cancer. miR-571 is known to play a role here and we identified Notch signaling as a putative function. Interestingly, Notch signaling has already been proposed as a drug target for fibrosis in other tissues. With the three case studies we demonstrated the necessity to use tissue-specific target gene information for miRNA function prediction.

To make our novel pathway analysis methodology publicly available, we systematically integrated 1) high-quality miRNA targeting data from TargetScan and CLIP-seq studies from StarBase v2 [5], 2) tissue specific gene expression from the latest version of EBI Expression Atlas [15] with 3) three major pathway databases KEGG [16], WikiPathways [17] and Reactome [18]. A graph database was used to store the data in a flexible manner and increase the query performance compared to relational data stores. The data backend and the corresponding pathway analysis methodology were integrated into miTALOS version 2 (v2), a user-friendly web application to identify pathways regulated by miRNAs in a tissue specific manner. With miTALOS v2 users can analyze multiple miRNAs together to account for combinatorial effects. MiTALOS v2 is complementary to other functional miRNA analysis tools such as miRGator [19] and ToppMir [20] and adds value with a tissue specific analysis of miRNA impact on signaling pathways. The integration of multiple new state-of-the art data sources increases the biological relevance of the results and a novel tissue filter allows every user to decipher complex miRNA functions.

## Results

### Tissue specific pathway enrichment

MiRNA target prediction tools and CLIP-seq based methods for target identification yield the full set of potential miRNA-mRNA interactions, i.e. all potential gene targets of a miRNA. However, different tissues and cell types have a characteristic gene expression signature and only a subset of genes are expressed in any cell under physiological conditions [12]. Thus, the function of miRNAs, which is exerted through repression of target genes, is tissue specific.

**Fig 1:**
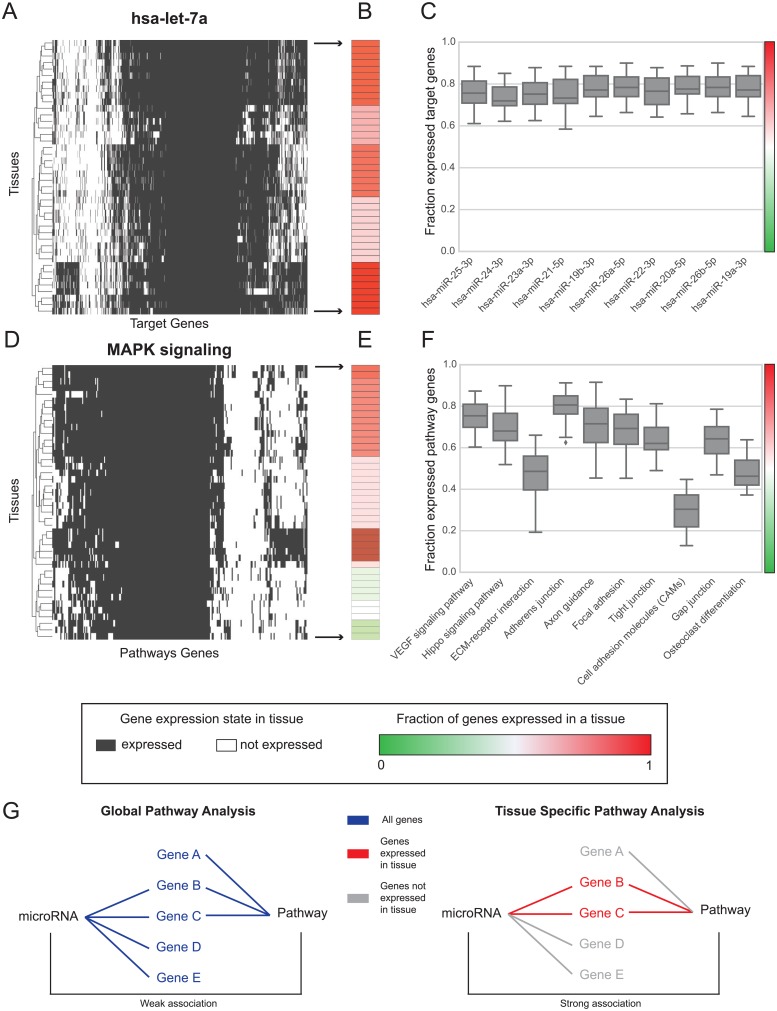
miRNA target genes and pathway genes are tissue specific. (A) Heatmap of all target genes of hsa-let-7a and their expression in 42 human tissues. Tissues are depicted in rows, genes in columns. (B) Fraction of target genes of hsa-let-7a expressed in each tissue, color coded in green (0) to red (1). (C) Fraction of target genes expressed in all tissues for 10 representative miRNAs. (D)-(F) Corresponding analysis for pathway genes. (G) Pathway analysis with the global set of miRNA targets and pathway genes (left). The miRNA and pathway have only few common genes (gene B, gene C) compared to the other pathway genes (gene A) and miRNA targets (gene D, gene E). When applying a tissue filter (right), genes not in the set of miRNA targets and not in the pathway are discarded. The association derived from the overlap is much stronger, indicating a tissue specific regulation of the pathway by the miRNA.

To learn about the tissue-specificities of miRNAs, we first analyzed the expression of all target genes of hsa-let-7a (TargetScan, see methods) in 42 human tissues from EBI Expression Atlas. The expression of target genes varied greatly between tissues (Fig 1A). To quantify the extent of tissue specificity of a miRNA, we calculated for each of the 42 tissues the fraction of target genes being expressed. The fraction is depicted in Fig 1B (color coded from green = 0 to red = 1). Fig 1C shows the respective distributions for ten representative miRNAs. Thereof, the median of target genes expressed in a tissue was 75%, with many tissues expressing only 60% target genes (Fig 1C). This is in line with studies showing tissue specific functions of miRNAs [13].

Next, we performed the same tissue-specificity analysis now only for genes of the same pathway. The pathway genes in well-described human MAPK signaling (KEGG) showed highly tissue specific expression (Fig 1D). Interestingly pathways showed a characteristic distribution of the fraction of expressed target genes when compared to miRNAs. Ten representative distributions across all 42 tissues are shown in Fig 1E. Some pathways (such as Cell adhesion molecules, Fig 1E) were more tissue specific than others, indicating highly tissue specific functions.

Having established that both miRNA and pathway associated genes have a characteristic gene expression signature across tissues, we next outlined the approach of standard miRNA pathway analysis methods. Typically the set of all miRNA targets are tested for over-representation in the set of all pathway genes (Fig 1F, left). This global analysis of all target and pathway genes will overlook miRNA-pathway associations with a small gene-overlap, while this gene-overlap may in turn be tissue-specific and, thus, functionally highly relevant. Pathway analysis tools that use all target genes to identify miRNA-pathway associations cannot capture tissue specific effects.

We thus propose a novel methodology for miRNA pathway analysis by using a tissue filter in order to increase the relevance of the association. If the target genes or pathway genes outside of the overlap are not expressed in a tissue, the relation of miRNA and pathway is much stronger (Fig 1F, right). Consequently, if the overlapping genes found in a miRNA-pathway association are not expressed in a tissue, the relation is discarded. The novel methodology calculates an enrichment of the target genes of a miRNA in all pathways of different pathway data sources. Significance of the associations is calculated with Fisher's exact test (see methods). Individually for each miRNA-pathway association test, we filtered for expression in a tissue by removing all miRNA target genes and pathway genes that are not expressed in this tissue. We thereby accounted for the highly tissue specific expression of many genes and seek to increase biological relevance of the pathway enrichment.

### Case study: microRNAs in liver disease

We analyzed miRNAs known to be involved in liver disease with our novel methodology to evaluate the power of tissue specific pathway analysis. We focused on miRNAs in hepatocellular carcinoma (HCC) and liver fibrosis. Both diseases involve uncontrolled proliferation of liver cells.

First, we analyzed miR-199a-3p and miR-199b-3p. Both miRNAs are up-regulated in some tumor types, such as ovarian cancer and breast cancer [21]. In HCC, conversely, both miRNAs have been shown to be down-regulated [22,23]. While the function of miR-199a-3p and miR-199b-3p is not fully defined, they target members of Raf/MEK/ERK signaling [23]. In general, inhibition of Raf/MEK/ERK signaling will limit proliferation of cells. Thus, downregulation of miR-199a-3p and miR-199b-3p might be a part of the regulatory changes leading to increased proliferation of HCC cells. These miRNAs have consequently been considered as therapeutic targets for treatment of HCC [24].

**Fig 2:**
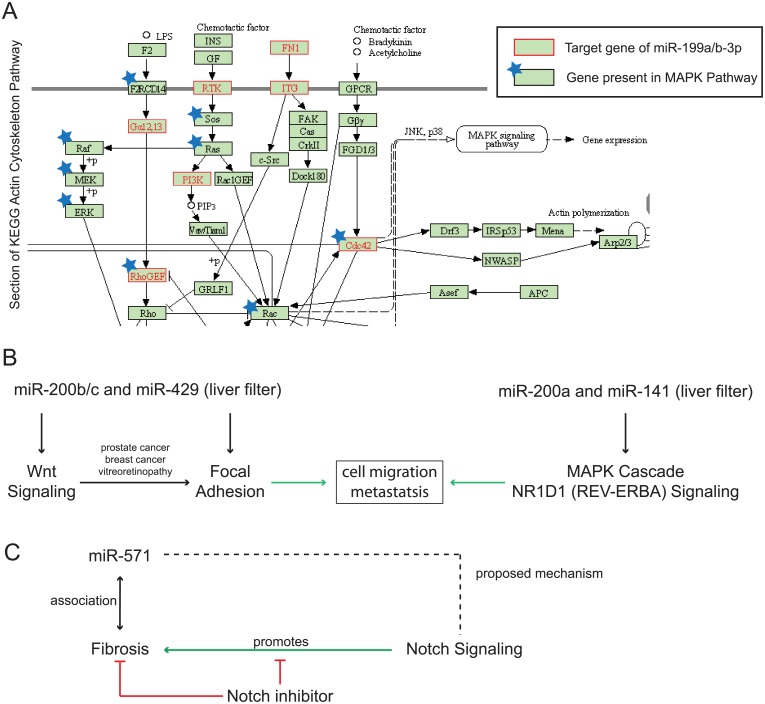
tissue specific enrichment of miRNAs in liver disease. (A) Targets of miR-199a/b-3p in the human KEGG Actin Cytoskeleton pathway (red). Only a section is shown, other parts are not targeted. Blue stars show genes also present in MAPK signaling. (B) Pathway analysis of miR-200 family using the liver filter. MiR-200b/c and miR-429 target Focal adhesion and Wnt signaling, pointing towards a regulatory interdependence in cancer formation. MiR-200a and miR-141 have different associated pathways but also target cancer related signaling. (C) MiR-571 is elevated in fibrosis and associated with notch signaling when using the liver filter. Notch inhibitors are in clinical studies for treatment of early stages of fibrosis.

When performing standard pathway analysis for miR-199a-3p and miR-199b-3p, no cancer-associated pathways were enriched (human, TargetScan). Using our methodology and the Illumina Body Map tissue filter for liver additionally identified two significantly associated pathways: Regulation of actin cytoskeleton (KEGG) and Regulation of Microtubule Cytoskeleton (WikiPathways) (Table 1). The miRNAs were previously not directly associated to regulation of the cytoskeleton, yet both pathways are fundamental for the processes of cell migration, EMT and metastasis. The regulation of actin cytoskeleton (KEGG) pathway overlaps with the MAPK signaling pathway from KEGG and includes several key components of Raf/MEK/ERK signaling (Fig 2A). The liver filter thus identified the known association of miR-199a-3p and miR-199b-3p with Raf/MEK/ERK signaling through associated regulatory pathways. Interestingly, the involvement of miR-199a/b-3p in cell migration and EMT has been described in other tissues [25,26].

**Table 1:** Pathway enrichment used in the case studies with liver filter.

source	Pathway	E	Corrected p-value	MP, Mn, Pn, U
*hsa-miR-199a*, *hsa-miR-199b-3p*				
wp	Regulation of Microtubule Cytoskeleton	3,814	0,034	4, 232, 18, 3982
kegg	Regulation of actin cytoskeleton	2,010	0,049	11, 225, 95, 3905
*hsa-miR-200b*, *hsa-miR-200c*, *hsa-miR-429*				
kegg	Focal adhesion	1,895	0,008	25, 593, 83, 3730
wp	Focal Adhesion	1,791	0,024	22, 596, 77, 3736
wp	Wnt Signaling Pathway	2,765	0,028	8, 610, 18, 3795
*hsa-miR-200a*, *hsa-miR-141-3p*				
wp	MAPK Cascade	3,194	0,047	5, 408, 15, 3910
reactome	NR1D1 (REV-ERBA) represses gene expression	19,095	0,017	2, 411, 1, 3924
*hsa-miR-571-3p*				
wp	Notch Signaling Pathway	9,844	0,000	3, 62, 20, 4069
kegg	Notch signaling pathway	8,945	0,001	3, 62, 22, 4067

Our novel methodology with tissue filter suggested a role of miR-199a/b-3p in cell migration, EMT and ultimately metastasis through regulation of cytoskeleton. The decrease of miR-199a/b-3p in HCC might increase metastatic potential in HCC. Indeed, a recent study indicates a role for miR-199a/b-3p in HCC proliferation [27].

Second, we investigated the miR-200 family consisting of two genomic clusters (miR-200b/c/miR-429 and miR-200a/miR-141) that was shown to be involved in EMT and cell migration [28]. The family has been described as a potential cancer therapy target [29]. The miRNAs of the miR-200 family are often analyzed together. Here, we look at specific functions of the two clusters to show the power of combined pathway analysis of multiple miRNAs. When performing pathway analysis with liver filter for miR-200b/c/miR-429 (Illumina Body Map, TargetScan, human) we identified significant associations with focal adhesion pathways from both KEGG and WikiPathways (Table 1). This finding clearly points towards an involvement in cell migration and EMT (Fig 2B). Interestingly, we also identified Wnt pathway (KEGG) (Table 1). As of today, there was no direct evidence reported for involvement of Wnt signaling in regulation of cell migration, EMT and metastatis in HCC. There was, however, evidence for a connection in other diseases such as breast cancer [30], vitreorenopathy [31] and prostate cancer [32].

Our novel methodology suggested new roles for miR-200b/c/miR-429 in HCC and a functional connection of Wnt signaling with cell migration and EMT (Fig 2B). Pathway analysis with liver filter (Illumina Body Map, TargetScan, human) for the other genomic cluster (miR-200a/141) identifies MAPK signaling and MAPK associated NR1D1-(REV-ERBA) pathway (WikiPathways) (Table 1). MAPK signaling was indeed elevated in HCC [33,34] and has been suggested as target for HCC treatment with success in mouse model [35] (Fig 2B). In summary, our novel methodology found specific HCC related functions for both genomic clusters of the miR-200 family. Analyzing the entire miRNA-200 family did not identify focal adhesion, Wnt or MAPK as significant results.

MiRNAs also play a role in liver fibrosis [36] but are in general less well studied in this disease context. There is only few functional evidence or mechanistic insight into the role of miRNAs in fibrosis. This represents an interesting example for the primary use case of our novel methodology: To generate new hypotheses and filter candidate miRNAs to be tested in the wet lab. The serum levels of miR-571 were found increased in cirrhosis (the final stage of fibrosis) and miR-571 has been suggested as a biomarker [37]. With our pathway analysis, we identified Notch signaling (KEGG) as target of miR-571 with liver filter (Illumina Body Map, human, TargetScan) (Table 1). Interestingly, Notch signaling was shown to be over-active in fibrosis [38] and Notch inhibitors have been discussed as potential drugs for treatment of fibrosis [38,39]. As a result, our novel methodology suggests that miR-571 could potentially inhibit Notch signaling in liver tissue. Thus, miR-571 might be a potential therapeutic target in the context of fibrosis (Fig 2C). In summary, our updated novel methodology supported new functional hypotheses through tissue filtered pathway analysis.

### Data sources

In order to make the novel methodology publicly available, we first integrated several data sources on miRNA targeting, biological pathways and gene expression for both mouse and human. We downloaded and integrated computational target prediction data from TargetScan 6.2 [40] and miRanda [41]. We also added miRNA-target interaction data of CLIP-seq studies from StarBase v2 [5]. TargetScan contained the majority of mammalian miRNAs while miRanda and StarBase only represented a small subset (Table 1). Due to the limited availability of CLIP-seq studies, we still rely on target prediction data for many miRNAs. Pathway data was extracted from KEGG [16], Reactome [18] and WikiPathways [17]. Pathways in the Reactome database were structured in top-level pathways with smaller sub pathways. This lead to larger numbers of pathways overall compared to KEGG and WikiPathways (Table 1). To allow for a tissue-specific pathway analysis, we used baseline gene expression data for a total of 68 human and mouse tissues and cell lines from the latest EBI Expression Atlas [15]. Baseline expression data was based on reliable RNA-seq experiments and represents abundance levels in healthy tissue or cell lines. We integrated tissue data sets from 6 different expression studies (Table 2).

**Table 2:** Overview of data sets.

	Human	Mouse
**# miRNAs**		
TargetScan v6	1529	1322
Miranda	249	238
StarBase v2	383	296
**# Pathways**		
KEGG	295	291
Reactome	2224	1882
WikiPathways	293	160
**# Tissues**		
Mammalian Tissues	8	6
Illumina Body Map	16	-
ENCODE Cell Lines	18	-
Vertebrate Tissues	-	5
6 Mouse Tissues	-	6
Nine Mouse Tissues	-	9

### Database backend

Any system that integrates heterogeneous research data has to deal with two major challenges: I) Data has to be stored in a way that it can be queried efficiently and II) the data model must allow for easy updates for new releases of the underlying data sources.

Traditionally, SQL based relational database systems such as MySQL or PostgreSQL were the go-to solution for all data storage needs. In recent years however, new database technologies collectively termed noSQL (short for not-only SQL) were developed to cope with problems arising from big data. Such noSQL technologies have been used successfully in solutions for computational biology, especially in the field of NGS [42]. Among the diverse landscape of new database technologies, graph databases are particularly promising for biological data sets. They enable storing data natively as a property graph, i.e. nodes connected by edges with properties stored on both. Thus, they allow us to directly model biological systems as nodes representing molecular entities connected by edges representing their interaction. This leads to simple queries over multi-step paths through the interaction network and increased performance compared to JOIN operations in relational databases [43,44]. Since queries on biological data are usually centered on relationships between molecular entities (such as genes and miRNAs), graph database have a huge potential to improve data storage solutions. The key advantages are query performance and simple query syntax.

**Fig 3:**
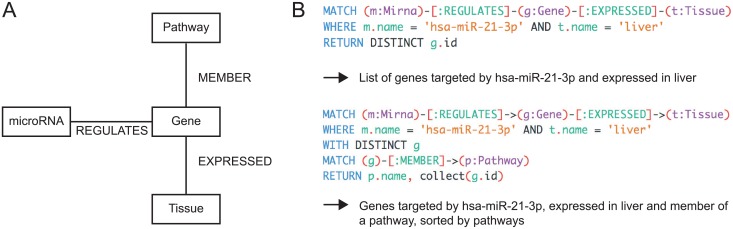
Database structure of miTALOS v2. (A) The miTALOS v2 dataset is stored in a graph database. The network structure allows for easy extension of the dataset. (B) The Cypher query language allows for simple queries on the network. With one query, the targets of a miRNA in a pathway can be accessed and filtered for tissue expression.

For our study, we used the graph database neo4j and developed a novel graph data model to integrate the data sources described above (Fig 3A). MiRNAs, genes, pathways and tissues were represented as nodes. MiRNAs were connected to genes with 'REGULATES' relationships, genes to tissues with 'EXPRESSED`relationships and genes to pathways with 'MEMBER' relationships. This data structure allowed us to e.g. query the target genes of a miRNA expressed in a tissue (Fig 3B, top) or the pathways in which the target genes are involved (Fig 3B, bottom).

Another challenge in studies based on integration of third party data sources is to keep up with data updates and new releases. Small, specialized data sources publish new versions on their own schedule and changes in one data source are not synchronized with others. Since neo4j is schema-less, changes of parts of the underlying data (e.g. miRNA targeting data for a single data source) and refactoring of the data structure (e.g. renaming of miRNAs) are easier to implement. We thus seek to regularly update our pathway analysis with new data sets especially focusing on NGS based data for miRNA targets and gene expression.

### miTALOS v2

In order to make our integrated, tissue specific pathway analysis available to the research community, we included the new analysis methodology and data backend in an update to our miTALOS web application.

**Fig 4:**
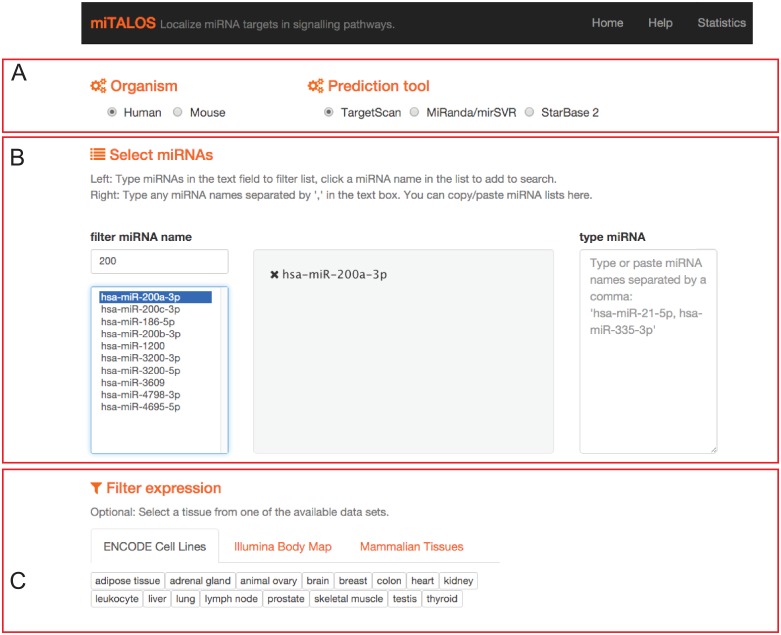
User interface of miTALOS v2. (A) The user starts by selecting the organism and miRNA prediction tool. Next, multiple miRNAs can be selected by filtering the list of available miRNAs (B). Lastly, a tissue filter can be applied by selecting an expression experiment and tissue or cell line (C).

MiTALOS v2 is a user-friendly tool to perform tissue specific pathway analysis for a set of miRNAs and tissues of interest (Fig 4). It is available at http://mips.helmholtz-muenchen.de/mitalos. The user can analyze miRNAs from mouse and human. The user begins by selecting the organism and miRNA prediction method (Fig 4A) and then selects one or multiple miRNAs (Fig 4B). The pathway analysis is carried out dynamically by calculating the pathway enrichment (see Methods) on all pathway data sources. If more than one miRNA is selected, the union of target genes will be used for the analysis. All target genes are counted once and no additional ranking is applied. MiTALOS v2 thereby captures the biological impact of co-targeting by multiple miRNAs. If the user selects a tissue filter, all gene sets (miRNA target genes and genes in pathways) are filtered for this tissue (Fig 4C). All results with a corrected p-value > 0.05 and *E* > 1 are presented in a sortable table and can be accessed with a user specific URL for one week. For KEGG pathways, the user can access a graphical representation of the pathway with highlighted miRNA targets by clicking on a pathway name.

If a tissue filter is used, miTALOS v2 displays the expression score of the selected miRNAs inaddition to the tissue specific pathway enrichment. The user can thereby assess the impact of the selected miRNAs under physiological conditions. The absolute expression score is extended by a rank of the selected miRNA among all miRNAs expressed in this tissue and the miRNA with the overall highest expression value. This allows estimating the relative importance of the selected miRNA in the analyzed tissue.

MiTALOS v2 is geared towards wet-lab researchers working with miRNAs. MiTALOS v2 was designed for scenarios where a set of miRNAs (e.g. from expression studies or literature research) has to be filtered to identify the most promising miRNAs for testing in wet-lab experiments. With the tissue filter, the user can analyze the supposed biological effect of miRNAs in the particular tissue or cell line the user is working on.

## Discussion

It has been established that miRNAs participate in almost all cellular processes but the functional impact of individual miRNAs and the precise mode of target gene regulation remains controversial. Consequently, the dynamic regulatory network of miRNAs and mRNAs under physiological conditions is not fully understood. One of the key issues in miRNA resarch is the identification and quantification of miRNA-mRNA interactions. While computational prediction methods and CLIP-seq approaches yield global sets of gene targets for individual miRNAs, they still suffer from lack of accuracy and fail to predict the regulatory landscape in-vivo.

One way to circumvent shortcomings in miRNA targeting data is to analyze the biological pathways which are incluenced by miRNAs. They can be considered a proxy for the miRNAs effect on biological processes and thus allow to classify miRNAs and generate new hypotheses. While pathway analyses have proven useful, they do not consider that most genes which are targeted by a miRNA or part of a pathway are not uniformly expressed across all cell types. The tissue specifity of miRNAs, which has been demonstrated extensively, is thus not taken into account.

By integrating tissue specific gene expression into our pathway analysis methodology, we seek to close this gap and improve the biological relevance of our miRNA-pathway associations. With our case studies, we recapitulated a common approach to generate new miRNA hypotheses for wet lab research: Based on prior knowledge, i.e. disregulation of several miRNAs in a disease context, the best candidates for experimental testing have to be identified. Our methodology aims at creating functional insight which is as specific as possible for the system studied by the user.

The distinctive feature of miTALOS v2 is the tissue specific pathway enrichment. Other pathway analysis tools, such as DIANA mirPath [45], do not account for this effect. MiTALOS v2 complements other methods for functional miRNA analysis. Tools analyzing the expression of miRNAs, such as MiRGator [19], aid in selecting the best miRNA candidates for a specific biological system. Ranking approaches, such as ToppMir [20], are used to limit the number of miRNAs based on preference for user-defined gene sets. MiTALOS v2 can be used in conjunction with these methods and adds a tissue specific perspective.

MiTALOS v2 includes CLIP-seq based miRNA targeting data from the StarBase database. CLIP-seq experiments generate the full set of target genes based on biochemically identified miRNA-mRNA interactions and likely produce more reliable targeting data than computational prediction. Several public resources, such as miRTarBase [46] and miRecords [47], collect miRNA targets validated in individual experiments. However, since these target sets contain only a potentially small subset of miRNA-mRNA interactions they would introduce a bias to the analysis and are thus not suitable for global pathway enrichment.

Next to TargetScan and miRanda, which were used in this study, there are several other miRNA target prediction tools. However, it is difficult to compare their performance due to the lack of a gold standard of known miRNA targets and systematic comparisons of target prediction tools generated inconsistent results [48–51]. TargetScan and miRanda were chosen based on their widespread use in the miRNA research community. If novel miRNA target data sources arise, the miTALOS v2 data can easily be integrated in miTALOS v2.

In general, the effect of a miRNA on its target genes cannot be quantified cell wide. The complexity of the miRNA-mRNA network was further increased when regulatory effects came into focus [52]. It was demonstrated that the total number of potential binding sites for a miRNA regulates its effect size. If the number of binding sites exceeds the number of miRNA molecules, mRNAs compete for binding to the miRNA and the regulatory impact decreases [53]. This has been subsumed under the concept of competing endogenous RNAs (ceRNAs). Recently, combined computational and experimental studies quantified these effects on a systems level [54]. Including these indirect effects into a pathway analysis presents a future direction for miTALOS v2. Here, using the relative expression levels of miRNAs and their target genes would allow to capture binding competition. However, more data on specific, quantitative effects will be necessary to devise a computational approach that properly describes the biological impact of competing RNAs.

When developing tools for the research community, the underlying data infrastructure is of pivotal importance. The state of the art, especially in research of post-transcriptional regulation, changes quickly and new methods for miRNA target identification might arise. We therefore developed a new database backend using neo4j, the leading graph database. It helps to integrate the numerous datasets used in miTALOS v2 and to keep up with new developments. The flexible backend also allows to integrate new aspects like lncRNAs as regulators of gene expression or disease specific expression profiles to extend tissue specific gene expression. New database technology is therefore instrumental in building tools which can adapt to the rapid generation of new research results.

In summary, our pathway analysis methodology and miTALOS v2 have been developed to generate testable hypotheses and to increase efficiency in experimental miRNA research.

## Methods

### Datasets

We integrated several data sources on miRNA targeting, biological pathways and gene expression in order to analyse tissue specific miRNA functions. For mouse and human, we offer computational target prediction data from the latest releases of TargetScan 6.2 [40] and miRanda [41]. We added miRNA-target interaction data of CLIP-seq studies from StarBase v2 [5] to the miTALOS v2 pathway analysis. Pathway data was extracted from KEGG, Reactome and WikiPathways. In order to analyze tissue specific pathway regulation, miTALOS v2 uses baseline gene expression data for 68 tissues and cell lines from the latest EBI Expression Atlas [15] for both mouse and human.

### Pathway analysis

We calculate an enrichment of miRNA target genes in pathways. For a miRNA *M* and Pathway *P* miTALOS v2 calculates a 2x2 cross table, where *MP* is the number of targets of *M* in *P*, *Pn* is the number of not targeted genes in *P*, *Mn* is the number of targets of *M* not in *P* and *U* is the union of all pathway genes and miRNA targets without *MP*, *Pn* and *Mn* (Table 3):

**Table 3:** 2x2 cross table.

	Pathway *P*	
miRNA *M*	MP	Mn
	Pn	U

An enrichment score *E* is calculated as the odds ratio of *M* and *P*:

E(M,P)=(MP/Pn)/(Mn/U)

*E* describes the dependence of variables *M* and *P*. *E* > 1 indicates an over-representation of targets of miRNA *M* in the pathway *P*. A p-value is calculated using Fisher’s exact test and results for multiple pathways are corrected using the Benjamini-Hochberg procedure [55].

To perform a tissue specific pathway enrichment, we remove all genes from *MP*, *Mn*, *Pn* and *U* that are not expressed in the analyzed tissue. We then calculate *E* as described above. A gene is considered expressed if its baseline expression value is > 0.5 (as defined in the EBI Expression Atlas).

When multiple miRNAs are selected, the union of target genes is used for the analysis.

### Database and webinterface

The integrated database backend is uses a neo4j graph database (v2.3.1). The miTALOS v2 frontend was developed with AngularJS 1.4.
